# Psychosocial correlates of disordered eating among adolescent athletes: a cross-sectional study

**DOI:** 10.1186/s40337-025-01500-x

**Published:** 2025-12-12

**Authors:** Amandine Franzoni, Jean-Philippe Antonietti, Simone Munsch, Nadine Messerli-Bürgy

**Affiliations:** 1 FAmily and DevelOpment research center (FADO), Institute of Psychology, University of Lausanne, Lausanne, Switzerland; 2 Department of Psychology, Clinical Psychology and Psychotherapy, University of Fribourg, Fribourg, Switzerland; 3 Food Research and Innovation Center, FRIC, Cluster Food and Mental Health/Psychology, University of Fribourg, Fribourg, Switzerland

**Keywords:** Disordered eating, Body dissatisfaction, Sociocultural pressures, Sports pressures, Athletes, Adolescence

## Abstract

**Background:**

Attempts to optimize nutritional intake and eating behaviors in young athletes can increase the risk of developing disordered eating (DE) during an athlete’s career. A multitude of factors influencing DE have been identified in the existing literature, encompassing general and sport-specific factors, and have been integrated into a conceptual model based on Petrie and Greenleaf’s work and additional research evidence in adults, but has not been proven in young athletes.

**Method:**

A total of 691 adolescent athletes (m/f: 276/415) aged 14–20 years, completed a set of online questionnaires assessing DE and different influential factors. Two separate structural equation models (SEMs) were tested: one for female athletes and one for male athletes.

**Results:**

The initial models showed poor fit; however, respecified models revealed key associations for each gender. Sociocultural pressures and their internalization primarily influenced body dissatisfaction in both genders, which contributed to DE. Additionally, emotion regulation difficulties and low self-esteem were linked to DE in female athletes, whereas sport weight pressures and negative mood were associated with DE in male athletes.

**Conclusion:**

Sociocultural pressures and body dissatisfaction critically affect DE in both female and male athletes, but etiological models for DE differ slightly between genders. If confirmed, prevention programs should be tailored accordingly.

**Supplementary Information:**

The online version contains supplementary material available at 10.1186/s40337-025-01500-x.

## Abstract (plain language summary)

Young athletes often try to improve their eating behaviors to boost performance. But this can sometimes lead to unhealthy eating behaviors. In this study, we wanted to better understand what puts adolescent athletes at risk for disordered eating. We asked 691 athletes aged 14 to 20 to complete an online questionnaire. We found that pressure from society — and how strongly athletes believed in these ideals — had a big impact on how they felt about their bodies. This was linked to disordered eating in both female and male athletes. However, some risk factors were different: for female athletes, low self-esteem and difficulty managing emotions were important. For male athletes, pressure about weight from their sport and negative mood played a bigger role. These results suggest that prevention programs should be tailored to the specific needs of female and male athletes.

**Supplementary Information:**

The online version contains supplementary material available at 10.1186/s40337-025-01500-x.

## Introduction

Nutrition plays a central role in athletes’ daily lives, but energy intake represents only one of several strategies used to regulate body weight and body composition in pursuit of performance goals [46]. Therefore, many athletes attach great importance to managing their weight or physical condition in order to meet sport-specific performance requirements [46]. Although such practices might be seen as a functional behavior in a sports context [27], the risk of crossing the thin line towards disordered eating is high. Disordered eating (DE) refers to a broad range of eating disorder symptoms that do not meet clinical diagnostic criteria, including occasional but repeated weighing, dietary restraint, intentional dehydration, use of weight control medication, purging behavior, self-induced vomiting, and excessive exercising [26, 49]. Disordered eating also refers to disturbances in perception, affect, and cognition towards one’s body, which can increase unhealthy behaviors [70]. Longitudinal studies have shown that the repeated use of such sports-related DE is associated with an increased risk of developing clinically relevant symptoms of an eating disorder (e.g., [65]). A recent systematic review reported that 19.23% of adolescent and adult athletes worldwide engage in DE [26], and the prevalence is even higher in studies with young elite athletes [21].

Several etiological models are useful to predict eating disorders (ED) and DE in the general population (e.g., the dual pathway model of Stice [60]). However, there is evidence that additional factors play a significant role in ED or DE of athletes that are not relevant to consider in the general population. Nevertheless, to date, there is only one model that is specific to athletes: the sociocultural model of Petrie and Greenleaf [43], which was initially developed in 2007 and most recently revised in 2020. This is a general theoretical model, not specific to any particular sport, level, or age group. Accordingly, it applies to both adult and adolescent athletes. According to this model [45], DE is determined by sport-related and individual factors. They assume that sociocultural and sports pressures related to appearance and weight play an important role in DE of athletes. According to them, sociocultural and sports pressures influence the internalization of body norms (i.e., the thin and muscular ideal, and a general desire to be physically attractive) in athletes, which results in greater body dissatisfaction and finally an increased risk for DE.

**Fig. 1:**
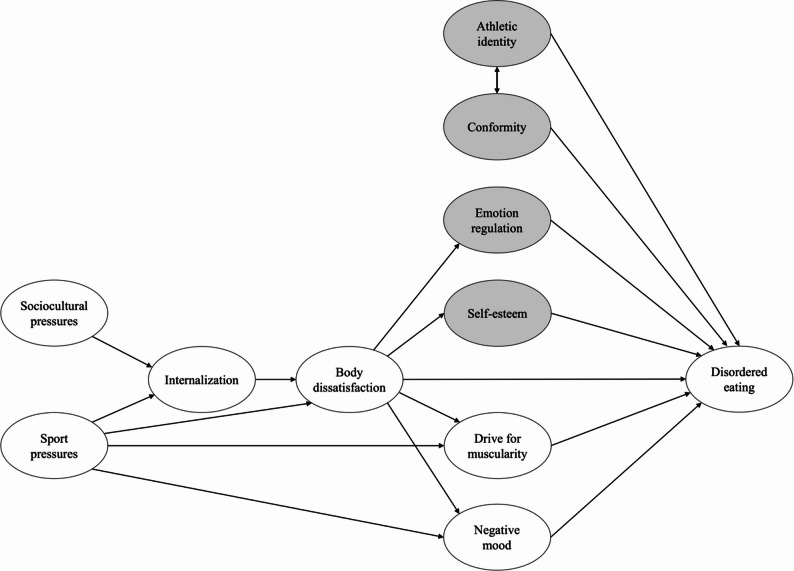
Conceptual model depicting the hypothesized pathways. Influencing factors added to the Petrie and Greenleaf model are shown in gray

According to their model (see Fig. 1), sports pressures also directly impact several other factors related to DE. Firstly, like etiological models of eating disorders in non-athletes (e.g., [61]), sports pressures have a direct impact on body dissatisfaction and the resulting negative affect, both of which increase the risk of DE. In addition, sports pressures increase the drive for muscularity, which is likely to be more expressed in athletes and increases DE symptoms [45]. Several of these associations between sport-related factors and DE, as proposed in Petrie and Greenleaf’s model (2012), have been supported by previous cross-sectional studies showing a consistent relationship with eating behavior in athletes. For instance, there is evidence that the repeated exposure to a sport-related body ideal conveyed by teammates and coaches is an additional pressure besides social media promoting a lean and thin body ideal which increases DE (e.g., [3, 14]). Young athletes are exposed to a sporting environment that conveys sport-specific norms (e.g., the body norm in long-distance running is thinness; [64]), which shape their body ideals – favoring either a muscular or thin physique depending on the sports category they choose. These constant pressures on athletes support the internalization of a thin or muscular body ideal (e.g., [3]). The discrepancy between the body ideal and the current body shape likely results in increased body dissatisfaction [39] which generates negative affect (e.g., anger, shame, guilt) and compensatory behavior to control body weight and muscularity, including dietary restraint, excessive exercising, purging, and substance use [54]. The choice of a specific compensatory behavior to achieve a specific body ideal may depend on the desired goal. For example, the pursuit of thinness – more common among women [18, 23] – is associated with increased purging behaviors, whereas the drive for muscularity – more prevalent in men – often leads to anabolic steroid use [30, 34, 71].

Petrie and Greenleaf’s [44] model is thought to be a work-in-progress approach to understanding the specific relationship between sports pressures and DE [45]. Given the growing body of evidence on the multiple influencing factors for DE in both athletes and non-athletes, the model needs to be extended accordingly. Consequently, we propose the necessity of certain adaptations to current knowledge in the development and maintenance of DE. While Petrie and Greenleaf’s original model incorporates restrained eating as a potential influencing factor, we suggest incorporating it into DE based on its role as a symptom of general eating pathology and eating disorders [2, 19] and related to the evidence of the validity of a transdiagnostic etiological and treatment approach [20]. In this study, we replaced the factor of negative affect with the longer-lasting construct of negative mood. This decision was informed by a recent systematic review of studies evaluating components of the Petrie and Greenleaf model [62], which found that six out of ten studies used measures of negative mood or negative emotions to operationalize the “negative affect” component. Conceptually, the two terms differ. Affect refers to short-term emotional reactions, whereas mood reflects a more enduring emotional state [53]. In practice, however, most self-report questionnaires tend to capture mood rather than momentary affect. Therefore, we opted to explicitly refer to “negative mood” to ensure greater conceptual and methodological alignment with both the present study and prior research.

Based on the existing literature on predictors and correlates of DE in both adolescent athletes and non-athletes (i.e., sociocultural and sport pressures, internalization of body ideals, body dissatisfaction, negative mood, and drive for muscularity), we propose to extend the original model of Petrie and Greenleaf by incorporating the following influencing factors: athletic identity, difficulties in emotion regulation, conformity to sport ethics, and self-esteem (highlighted in grey in Fig. 1). The extended model is organized according to the logic of a path model: variables are positioned from left to right based on whether they function conceptually as exogenous or endogenous variables. Factors without antecedent variables appear at the leftmost level, while the outcome variable (disordered eating) is placed at the rightmost level. The additional factors shown in grey were positioned accordingly: athletic identity and conformity to the sport ethic were included as direct predictors of DE, whereas self-esteem and emotion regulation difficulties were placed between body dissatisfaction and DE, reflecting their position in the conceptual sequence of the model. These additional factors are likely to better capture sport specific as well as general psychosocial mechanisms contributing to DE in adolescents. First, cross-sectional studies in adolescent athlete populations have shown that DE are associated with the subjectively experienced belonging of the young athletes to their sport discipline and, therefore, their adherence to sport, the specific ethic norms, or the athletic identity (e.g., [7, 25, 66]). The sport ethic refers to a set of cultural norms that delineate the behaviors and values associated with being perceived as a “true athlete”, including making sacrifices for the sport, striving for distinction, accepting risk and playing through pain, and refusing to accept limits [31]. While a certain degree of conformity to these norms is often necessary for success in competitive sports, an unquestioning acceptance or rigid commitment to these norms may be associated with unhealthy behaviors, including training while injured [37], substance use [67], as well as DE (e.g., [15, 46, 69]). In addition, the primary identification of oneself with an athletic status may be associated with young athletes placing a higher value on their sport [66] than on other important developmental tasks. Additionally, athletic identity and conformity to the sport ethic are closely intertwined and may be associated with one another [6, 15, 29].

Second, we propose integrating two general psychological factors in this model known to influence DE in non-athlete adolescents: self-esteem and emotion regulation difficulties. Both factors had not been considered in the model so far, but previous studies have shown that emotion regulation difficulties can mediate the relationship between body dissatisfaction and DE in both adolescents [13, 40, 57] and young adults [17]. Similarly, low self-esteem has been linked to both body dissatisfaction and DE [5, 8]. Together, these factors have consistently been identified as important correlates of disordered eating and of clinical eating disorders in non-athletes [18, 22, 35, 47]. Despite their relevance in the general population, these two variables remain understudied in adolescent athlete populations, warranting their inclusion in our extended model.

To date, several studies have tested and confirmed different parts of Petrie and Greenleaf’s (2012) model, but findings are inconsistent (see [62] for a review) and only Chatterton et al. [12] have evaluated the full model in a cross-sectional sample of 698 U.S. male collegiate athletes (*M*_age_ = 19.87 years). They confirmed that sports pressures were associated with the internalization of body ideals, body dissatisfaction, dietary restraint, negative affect, and drive for muscularity. Additionally, they found a link between sociocultural pressures and the internalization of body ideals. Internalization was associated with high body dissatisfaction, which in turn was linked to dietary restraint and negative affect. They also confirmed that drive for muscularity, dietary restraint, body dissatisfaction, and negative affect were directly associated with DE in these male collegiate athletes. Further, they identified an additional link between sociocultural pressures and negative affect, but the relation between body dissatisfaction and drive for muscularity could not be confirmed. Beyond that, no study has examined the model in its entirety among both male and female athletes, younger athletes, different sport categories, or competitive levels. Furthermore, none of the previous studies have tested this model while simultaneously integrating factors recently identified in non-athlete samples (i.e., self-esteem and emotion regulation) nor in an athlete population (i.e., conformity to sport ethic norms and athletic identity). The aim of the present study was therefore to test an extended version of Petrie and Greenleaf’s theoretical model [45], incorporating these new factors, in a cross-sectional sample of adolescent athletes (both male and female) from various sports and competition levels in Switzerland, with the intention of evaluating a general model applicable across sport types rather than developing discipline- or level-specific pathways.

Based on the original model, we hypothesized that in young athletes (1) high levels of sociocultural and sports pressures are related to higher levels of internalization of body ideals; (2) high levels of internalization are related to more body dissatisfaction; (3) sports pressures are related to higher levels of negative mood, more body dissatisfaction, and more drive for muscularity; (4) high levels of body dissatisfaction are related to higher levels of negative mood and more drive for muscularity; and (5) that high levels of drive for muscularity, negative mood, and body dissatisfaction are related to higher levels of disordered eating.

In addition to these core hypotheses, and based on recent research, we extended the model to include four additional influencing factors of DE in young athletes. Specifically, we hypothesized that (6) higher levels of body dissatisfaction are related to lower self-esteem, and more difficulties in emotion regulation; (7) lower self-esteem and greater difficulties in emotion regulation are, in turn, associated with higher levels of DE. Further, we hypothesized that (8) athletic identity and conformity to the sport ethic norms are positively correlated; and that (9) both higher athletic identity and levels of conformity are related to higher levels of DE. Finally, we expected no differences in the model structure between male and female athletes.

## Method

### Participants and procedures

Participants were recruited between September 2023 and March 2024 through advertisements in sports clubs, medical centers, and through social media in different French-, German-, and Italian-speaking areas of Switzerland. Participants were eligible if: (1) aged between 14 and 20, (2) officially registered in a sports club, (3) participated in organized regional, national, or international competitions and club training (i.e., with the presence of at least one coach), and (4) fluent in one of the official languages (French, German, or Italian). Individuals with a current injury (for more than one month), or with a severe mental or physical health problem (i.e., requiring intensive treatment) were all excluded from study participation. Participants with a current or past eating disorder were not excluded. Only conditions requiring intensive treatment or situations that could compromise participant safety were considered as exclusion criteria. The exclusion criteria were specified in the participant information sheet and were assessed in the demographic questionnaire at the beginning of participation. Those answering “yes” to either question were excluded. These criteria were chosen for ethical and methodological reasons, including avoiding risks for participants and preventing biased responses on measures assessing behaviors over the past 28 days. The study protocol was approved by the ethics committee of the study protocol was approved by the ethics committee of the University of Lausanne, Switzerland (E_SSP_072023_00001), and the study was conducted according to the Declaration of Helsinki.

All participants individually accessed the online survey, where they were asked to complete a set of questionnaires anonymously after giving their informed consent. According to Swiss law at the time, parental consent was not a prerequisite for minors. The survey was accessible through the Qualtrics platform, and the average completion time was 45 min. At the end of the survey, participants could take part in a raffle for shopping vouchers as reimbursement. Data quality was monitored, and implausible or inconsistent entries (e.g., extremely short completion times or repeated response patterns) were removed during data cleaning to reduce the likelihood of illegitimate participation.

A total of 1097 athletes participated in this survey and 691 participants (m/f: 40%/60%) aged between 14 and 20 years completed more than 50% of the questionnaires and were therefore considered for data analysis. Descriptive statistics among demographic variables are shown in Table 1. Within this final sample, most female athletes (57%) engaged in weight-sensitive sports (i.e., gravitational (e.g., ski jumping), weight-dependent (e.g., boxing), aesthetic (e.g., synchronized swimming), or endurance sports (e.g., rowing); [1, 63]), while the majority (66%) of male athletes engaged in non-weight-sensitive sports (i.e., ball games (e.g., basketball), power (e.g., powerlifting), technical (e.g., fencing), or motor sports (e.g., motorcycling)). Participants competed at different levels, where the most common was national level (m/f: 47%/30%), followed by regional/interregional levels (m/f: 36%/49%), international levels (m/f: 14%/16%/), and a few could not be assigned to one level (m/f: 3%/5%).

**Table 1:** Descriptive statistics of demographic and study variables

	Male sample	Female sample	Possible range
*n (%)*	276 (39.9)	415 (60.1)	
Age (years) *M(SD)*	16.51 (1.83)	16.31 (1.78)	
Sport category *n*(%)			
Ball games	170(61.6)	156(37.6)	
Aesthetic sports	9(3.3)	106(25.5)	
Power sports	3(1.1)	4(1.0)	
Technical sports	7(2.5)	19(4.6)	
Endurance sports	65(23.5)	107(25.8)	
Weight-dependent sports	12(4.3)	13(3.1)	
Gravitational sports	9(3.3)	10(2.4)	
Motor sports	1(0.4)	0(0.0)	
Sport level *n*(%)			
International	38(13.8)	65(15.7)	
National	130(47.1)	125(30.1)	
Interregional	46(16.7)	96(23.1)	
Regional	52(18.8)	109(26.3)	
Other	10(3.6)	20(4.8)	
Sport experience *n*(%)			
< 1 year	16(5.7)	21(5.1)	
1 to 5 years	78(28.3)	120(28.9)	
> 5 years	182(66.0)	274(66.0)	
Training (hours) *M(SD)*	8.96(4.99)	9.00(5.66)	
BMI (kg/m^2^) *M(SD)*	21.16(2.90)	20.84(2.41)	
Study variables *M(SD)*			
Disordered eating (DE)	0.56(0.75)	0.98(0.94)	0–6
Sport weight pressures	2.11(0.78)	2.15(0.91)	1–6
General sociocultural pressures	2.05(0.85)	2.32(0.90)	1–5
Internalization of body ideal	3.42(0.87)	3.63(0.82)	1–5
Body dissatisfaction	3.01(2.17)	2.58(1.61)	0–14
Drive for muscularity	3.47(1.31)	2.73(1.13)	1–6
Conformity	2.81(0.57)	2.61(0.58)	1–4
Athletic identity	5.22(1.13)	5.24(1.00)	1–7
Self-esteem	1.77(0.53)	2.16(0.62)	1–4
Difficulties in emotional regulation	1.93(0.72)	2.41(0.82)	1–5
Negative mood	0.60(0.51)	1.00(0.59)	0–3

*BMI* body mass index measured by self-reported height and weight

### Measures

The supplementary material (Table S1) contains all the information regarding the reliability and validity of the measures, as well as the versions that were used.

#### Disordered eating (DE)

The Eating Disorder Examination Questionnaire (EDE-Q; [19]) was used to assess *disordered eating* over the past 28 days. However, to avoid conceptual overlap between the body dissatisfaction variable and the *weight and shape concerns* subscale of the EDE-Q, a total of 16 items related to *disordered eating* were selected and coded according to Wyssen et al. [73]. Items include the subscales *eating concerns* (items 7, 9, 19, 20, 21; ɑ_female_ = 0.82, ɑ_male_ = 0.69), *restraint* (items 1, 2, 3, 4, 5; ɑ_female_ = 0.85, ɑ_male_ = 0.68) and *inappropriate compensatory behaviors* (e.g., frequency of binge-eating, self-induced vomiting, laxative use, and excessive exercising; items 13–18; ɑ_female_ = 0.71, ɑ_male_ = 0.41). The frequency of these behaviors is assessed on a seven-point response scale ranging from 0 (*0 days*) to 6 (*every day*). Mean levels are calculated for each subscale, with higher scores indicating more eating concerns, more restraint behaviors, and more inappropriate compensatory behaviors representing more DE.

#### Sport weight pressures

The 16-item *Weight Pressure in Sport in female athlete* (WPS-F; [50]) and the 12-item *Weight Pressure in Sport in male athlete* (WPS-M; [24]) were administered. Translation for the missing language version (German, Italian, and partially into French) was prepared according to the standards of Corbière & Fraccaroli [16]. In these questionnaires, individuals are asked to indicate the frequency of experienced pressure on a six-point Likert scale, ranging from 1 (*never*) to 6 (*always*). The female version comprises two factors: *weight pressures from coaches/team/sport/weight limits* (ɑ = 0.89) and *self-consciousness of weight/appearance* (ɑ = 0.81). The male version comprises three subscales: *teammate pressures about weight* (ɑ = 0.80), *importance of body weight and appearance* (ɑ = 0.52), and *pressure about weight and body due to the sports uniform* (ɑ = 0.73). Mean values are calculated, and higher scores indicate more perceived pressure.

#### General Sociocultural pressures

The subscales regarding appearance-related pressures of the *Sociocultural Attitudes Towards Appearance Questionnaire-4 revised for female* (SATAQ-4R-Female, 16 items) and the *SATAQ-4R-Male* (20 items; [55]) were used. Both versions were translated into German and French by the authors as there was no translated version available. In accordance with the original, the two versions differ slightly in terms of subscales with the female version comprising three subscales (i.e., *Pressures by peers/significant others* (ɑ = 0.91), *Pressures by the family* (ɑ = 0.87), and *Pressures by media* (ɑ = 0.94)), and the male version only two factors (*Pressures by family/peers/significant others* (ɑ = 0.93), *Pressures by media* (ɑ = 0.95)). Individuals respond on a five-point Likert scale, ranging from 1 (*definitely disagree*) to 5 (*definitely agree*). The scores for each subscale are obtained by averaging the items, with higher scores indicating greater pressures.

#### Internalization of body ideal

The subscales regarding *internalization of the thin ideal* (ɑ_female_ = 0.82, ɑ_male_ = 0.81), of the *muscular ideal* (ɑ_female_ = 0.88, ɑ_male_ = 0.89), and of a *general desire to be physically attractive* (ɑ_female_ = 0.84, ɑ_male_ = 0.81) of the SATAQ-4R-Female (15 items) and the SATAQ-4R-Male (8 items; [55]) were used. Individuals respond on a five-point Likert scale, ranging from 1 (*definitely disagree*) to 5 (*definitely agree*). The scores for each subscale are obtained by averaging the items, with higher scores indicating greater internalization.

#### Body dissatisfaction

The *Body Image Matrix of Thinness and Muscularity – Female Bodies* (BIMTM-FB; [59]) and the *Body Image Matrix of Thinness and Muscularity – Male Bodies* (BIMTM-MB; [4]) are figure rating scales containing two orthogonal dimensions, body fat and muscularity. Each figure is composed of eight individual silhouettes, with the body fat and musculature increasing in small increments. Consequently, the matrix comprises 8 × 8 bodies, resulting in a total of 64 figures, each numbered from 1 to 64. The range of body shapes depicted includes individuals with very low body fat, individuals with very high body fat, and individuals with varying degrees of muscularity. Individuals are asked to indicate the number of the figure that best corresponds to (a) their actual, (b) their felt, and (c) their ideal body image. Body dissatisfaction is calculated based on the Manhattan distance approach between (1) the actual and ideal body image and (2) the felt and ideal body image. Higher scores indicate higher levels of body dissatisfaction.

#### Drive for muscularity

The *Drive for Muscularity Scale* (DMS; [38] is a 15-item scale that allows assessment of individuals’ desire for a more muscular body including muscularity-oriented body image (ɑ_female_ = 0.89, ɑ_male_ = 0.92) and behavior (ɑ_female_ = 0.77, ɑ_male_ = 0.80). For the French version the shortened version of Chaba et al. [11] was complemented by a translation of the remaining five items. Individuals respond on a six-point Likert scale, ranging from 1 (*never*) to 6 (*always)*. A higher score indicates a greater drive for muscularity.

#### Conformity

The degree to which athletes conform to the sport ethic was assessed by the *Conformity to the Sport Ethic Scale* (CSES; [42]). The CSES was translated into German and Italian. This 20-item questionnaire includes three factors: (1) *striving for distinction* (ɑ_female_ = 0.79, ɑ_male_ = 0.81), (2) *self-sacrifice* (ɑ_female_ = 0.78, ɑ_male_ = 0.76), and (3) *refusing to accept limits* (ɑ_female_ = 0.82, ɑ_male_ = 0.71). Individuals respond on a four-point Likert scale, ranging from 1 (*strongly disagree*) to 4 (*strongly agree*). Mean levels are calculated, with a higher score indicating stronger conformity with the sport ethic.

#### Athletic identity

The degree to which athletes identify with the athlete role was assessed using the 7-item *Athletic Identity Measurement Scale* (AIMS; [9]). The AIMS is composed of three subscales: (1) *social identity* (ɑ_female_ = 0.65, ɑ_male_ = 0.76), (2) *exclusivity* (ɑ_female_ = 0.81, ɑ_male_ = 0.85), and (3) *negative affectivity* (ɑ_female_ = 0.53, ɑ_male_ = 0.66). Individuals respond on a seven-point Likert scale, ranging from 1 (*strongly disagree*) to 7 (*strongly agree*). Higher scores indicate more athletic identification.

#### Self-esteem

The *Rosenberg Self-Esteem Scale* (RSES; [51]) is assessing global self-esteem (ɑ_female_ = 0.90, ɑ_male_ = 0.86). The questionnaire consists of 10 items and responses are given on a four-point Likert scale, ranging from 1 (*strongly agree*) to 4 (*strongly disagree*). Higher scores indicate lower levels of self-esteem.

#### Difficulties in emotional regulation

The 18-item *Difficulties in Emotion Regulation Scale – Short Form* (DERS-SF; [28]) was used to measure difficulties in emotional regulation, in six subscales; (1) *strategies* (ɑ_female_ = 0.77, ɑ_male_ = 0.73), (2) *nonacceptance* (ɑ_female_ = 0.81, ɑ_male_ = 0.79), (3) *impulse* (ɑ_female_ = 0.92, ɑ_male_ = 0.89), (4) *goals* (ɑ_female_ = 0.89, ɑ_male_ = 0.90), (5) *awareness* (ɑ_female_ = 0.76, ɑ_male_ = 0.76), and (6) *clarity* (ɑ_female_ = 0.66, ɑ_male_ = 0.70). Individuals respond on a five-point Likert scale, ranging from 1 (*almost never*) to 5 (*almost always*) where mean levels are calculated, with higher scores indicating more difficulties to regulate one’s emotions.

#### Negative mood

*The 9-item Patient Health Questionnaire-9* (PHQ-9; [58]) was utilized in this study as an indicator of negative mood (ɑ_female_ = 0.85, ɑ_male_ = 0.85). The participants responded on a four-point Likert scale, ranging from 0 (*not at all*) to 3 (*nearly every day*). The computed means indicated higher levels of negative mood.

### Statistical analysis

As a preliminary analysis, exploratory factor analysis was conducted to test the hypothesized construct of each latent variable. As anticipated, the negative mood and self-esteem variables were unidimensional in both female and male samples. To ensure that these measures are not the sole indicators of their hypothesized latent variables, each construct was parceled (into three indicators for self-esteem and negative mood), following the approach of Little et al. [36].

To test the proposed model, structural equation modeling [33] was performed in the R environment (v4.2.2; [48]) using the *lavaan* package (v0.6–17; [52]). First, confirmatory factor analysis was used to establish the measurement model, assessing the relationships of the measured variable to the latent variable. All measured variables (using the subscales or parcels) with poor loading were excluded from further analysis. When only one variable remained for a latent variable, this variable was parceled [36]. Then, the structural model was tested to determine the strength and significance of the proposed pathways among the latent variables. All models were tested using the maximum likelihood estimator with robust standard errors (MLR) to account for non-normality in the data. Missing data (0.12% in female sample, 0.47% in male sample) were handled using the full-information maximum likelihood (FIML) method. Several statistical indices were considered to evaluate the model fit, using Kline’s recommendations [33]: the robust comparative fit index (CFI), the robust Tucker Lewis index (TLI), the robust root mean square error of approximation (RMSEA), and the robust standardized root mean square residual (SRMR). The indices collectively ensured a comprehensive evaluation of the model fit. Akaike Information Criterion (AIC) and Bayesian Information Criterion (BIC) were used as parsimony indices to test differences between models. In general, values that are indicative of good fit include CFI and TLI greater than 0.90, RMSEA under 0.08, and SRMR under 0.08 [32]. Lower values of AIC and BIC indicate a better model fit than compared models [10]. As the questionnaires varied between genders and as recommended by [62], we conducted all the statistical analyses separately in the two sample populations.

## Results

### Factors related to DE in young female athletes

Estimation of the measurement model (see Appendix A for more details) revealed a poor fit, and therefore the following variables were dropped: Internalization – muscular (INT1), Emotional regulation – Conscience (ER6), and Drive for muscularity – Muscularity behavior (DM2). Furthermore, Drive for muscularity – Muscularity-oriented body image (DM1) was parceled into three indicators, and the revised measurement model demonstrated finally a good overall fit ($\chi^2$ (380) = 896.989, CFI = 0.92, TLI = 0.90, RMSEA = 0.059 [90% CI: 0.054–0.064], SRMR = 0.073, AIC = 30309.480, BIC = 30901.637), with factor loadings ranging from 0.51 to 0.96, *ps* < 0.001.

**Fig. 2:**
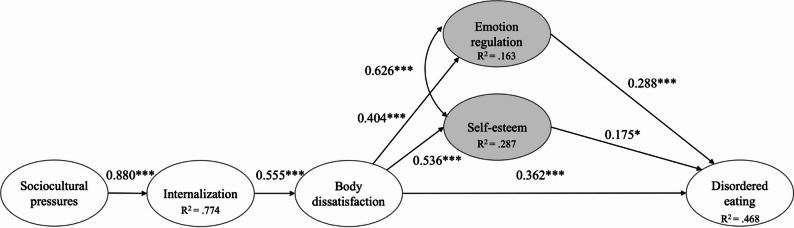
Respecified structural model for the female sample. The parameter estimates are standardized coefficients. *p <.05, **p <.01, ***p <.001

Analyses revealed a poor fit of the hypothesized model (see Fig. 1) with the data ($\chi^2$(412) = 1142.800, CFI = 0.89, TLI = 0.87, RMSEA = 0.067 [90% CI: 0.062–0.072], SRMR = 0.083, AIC = 30506.772, BIC = 30970.024). Therefore, this model was respecified following guidelines outlined by [74]. First, nonsignificant pathways were dropped. Second, based on modification indices, the covariance between self-esteem and emotion regulation was added to this model. The resulting respecified model (see Fig. 2) provided an acceptable fit to the data ($\chi^2$(127) = 403.716, CFI = 0.92, TLI = 0.91, RMSEA = 0.076 [90% CI: 0.068–0.085], SRMR = 0.115, AIC = 17372.892, BIC = 17622.735), and all pathways were significant (*p*s < 0.05). AIC and BIC values for the respecified model were the lowest, thereby further reinforcing the robustness of the final model. All indirect effects were statistically significant: between sociocultural pressures and DE through emotion regulation (β = 0.07, *p* =.003), through self-esteem (β = 0.05, *p* =.016), and through body dissatisfaction (β = 0.21, *p* <.001).

The final model revealed associations between sociocultural pressures and internalization of body ideals, internalization and body dissatisfaction, and body dissatisfaction with various factors, including self-esteem, emotion regulation, and DE. Further, these two new factors of the model (i.e., emotion regulation and self-esteem) were both associated with DE.

### Factors related to DE in young male athletes

For male athletes, the initial measurement model (see Appendix A for more details) had a poor fit with the data and therefore the following variables were dropped: Internalization – thin/low body fat (INT2), Emotional regulation – Conscience (ER6), and DE – Compensatory behaviors (DE3). Furthermore, based on modification indices, a covariance adjustment between two variables of emotion regulation (ER3 and ER4) was incorporated. After modifications, the revised measurement model demonstrated a good overall fit ($\chi^2$(471) = 852.487, CFI = 0.92, TLI = 0.90, RMSEA = 0.054 [90% CI: 0.074-0.060], SRMR = 0.062, AIC = 21511.894, BIC = 22083.917), with factor loadings ranging from 0.46 to 0.99, *ps* < 0.001.

**Fig. 3:**
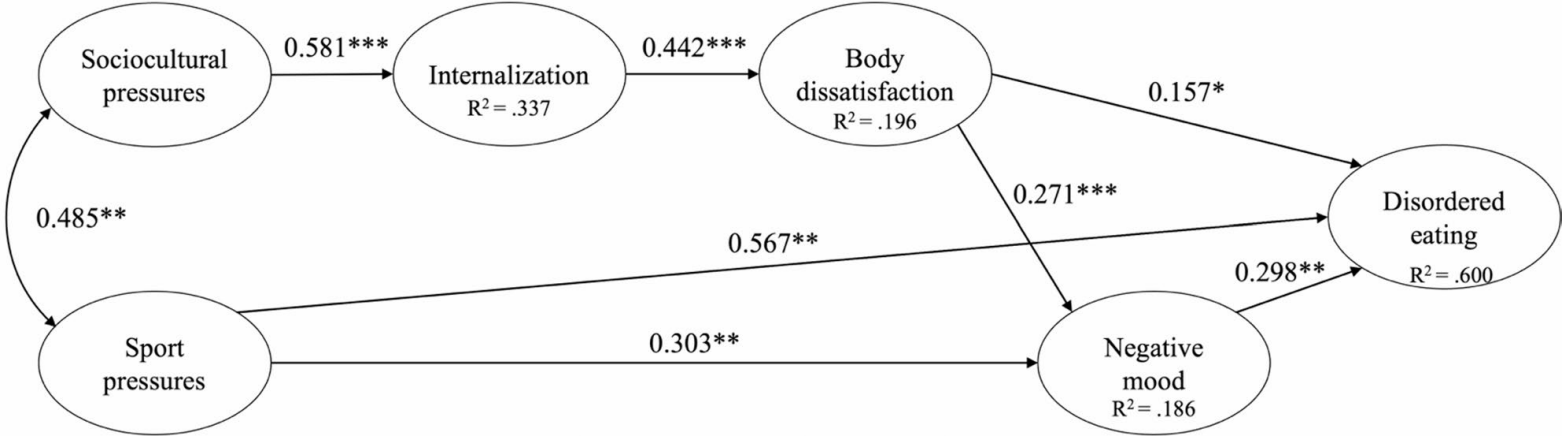
Respecified structural model for the male sample. The parameter estimates are standardized coefficients. *p <.05, **p <.01, ***p <.001

Further, the hypothesized model had a poor fit with the data ($\chi^2$(503)) = 1249.615, CFI = 0.85, TLI = 0.83, RMSEA = 0.071 [90% CI: 0.066–0.077], SRMR = 0.128, AIC = 21819.924, BIC = 22276.095). The model was respecified, initially by dropping nonsignificant pathways. In a second step, based on modification indices and consideration of existing theory, the pathway between sport pressures to DE and a covariance between sport pressures and sociocultural pressures were added to the model. The resulting respecified model (see Fig. 3) provided a good overall fit to the data ($\chi^2$(96) = 221.115, CFI = 0.93, TLI = 0.91, RMSEA = 0.069 [90% CI: 0.057–0.082], SRMR = 0.074, AIC = 11009.403, BIC = 11212.146), and all pathways were significant (*p*s < 0.05). AIC and BIC values for the respecified model were the lowest, thereby further reinforcing the robustness of the final model. The indirect effects between sociocultural pressures and DE through body dissatisfaction (β = 0.03, *p* =.045) and sport pressure to DE through negative mood (β = 0.13, *p* =.023) were statistically significant. No indirect effect was found between sociocultural pressures to DE through negative mood (β = 0.15, *p* =.066).

The final model revealed associations between sociocultural pressures and internalization of body ideals, internalization and body dissatisfaction. Further body dissatisfaction was associated with negative mood and with DE. In addition, negative mood was related to DE. Besides this, there was a significant relation of sports pressures with sociocultural pressures, with negative mood and with DE.

## Discussion

This study aimed to investigate an extended version of Petrie and Greenleaf’s sociocultural model of disordered eating [45] by incorporating additional variables currently discussed in the field of eating disorder research in sports and beyond (i.e., conformity to the sport ethic norms, athletic identity, self-esteem, and emotion regulation) in a younger mixed-gender sample of athletes. Due to slight differences in the assessment of the concepts of general and sport weight pressures, internalization of body ideals, and body dissatisfaction in both genders, data were analyzed separately. The findings supported several theoretically proposed associations while also revealing distinct gender-specific patterns.

Across both genders, structural paths between sociocultural pressures, internalization of body ideals, body dissatisfaction, and DE were supported in this sample of adolescent athletes, consistent with previous studies (e.g., [3, 12, 68]). In both models, higher levels of pressure from family, peers, media, and significant others regarding appearance were associated with higher internalization of these beauty and body standards in adolescent athletes. This internalization, in turn, was associated with greater body dissatisfaction and increased DE. Although the cross-sectional design does not allow conclusions about directionality, these patterns suggest that these factors may already co-occur during early adolescence.

Among female athletes, the two factors ‘emotion regulation and self-esteem’ were both associated with DE, whereas this was not the case in the male model. These findings for the female sample are in line with prior research emphasizing the importance of transdiagnostic factors for DE in adolescent and adult populations (e.g., [13, 35, 47], [56]). The covariance observed between self-esteem and emotion regulation suggests that these constructs may represent a broader emotional dysfunctioning profile (with generally low self-esteem and difficulties in regulating one’s emotions), both relevant to DE vulnerability. One potential explanation for why emotion regulation and self-esteem were observed only among female athletes lies in the descriptive differences between the two groups. In the male sample, both emotion regulation difficulties and self-esteem showed lower mean levels and reduced variability compared to the female sample. Such differences in distribution can reduce the predictive power of these variables in structural equation modeling.

In contrast, among male athletes, sport pressures and negative mood played an important role in the model. Sports pressures were directly associated with both negative mood and directly and indirectly (through negative mood) associated with DE. Although directionality cannot be inferred, this pattern suggests that performance-related demands may be more proximally related to emotional distress provoking negative mood and disordered eating in this group. Besides this, there was an association between sports pressures and sociocultural pressures in the male sample, with consistent covariance levels observed as in a previous study [12], representing a reciprocal reinforcement of societal and sport-specific appearance expectations rather than both operating independently.

Interestingly, sport weight pressures were not significantly associated with the internalization of body standards in either gender, aligning with [3]. A recent study by Pallotto et al. [41] that included female collegiate athletes also found that sport-specific weight pressures were not associated with internalization of ideals of muscularity and thinness, nor with body dissatisfaction. This may suggest that, within adolescent athlete samples, internalization of body ideals and body dissatisfaction may be more closely linked to broader sociocultural pressures than to sport-specific weight pressures [41, 45].

Contrary to our expectations, based on prior literature (e.g., [12, 15, 46, 69, [75]), all other factors such as drive for muscularity, athletic identity, and conformity to the sport ethic were not significantly associated with DE in either gender. One possible explanation is that these factors may be more prominent in elite sport environments, where performance demands, identity investment, and adherence to sport norms are typically heightened. Another possibility is that athletic identity and conformity to the sport ethic may be less pronounced among younger athletes, who are still early in their athletic career and may not yet fully have internalized these norms or developed a strong sense of athletic identity, particularly given that adolescence is a critical period for identity development. In the present sample, which included athletes from various sports and competition levels, the influence of these variables may have been diluted or context-dependent, and therefore less likely to emerge as significant. Unfortunately, we were unable to compare the mean levels of these variables with existing findings in adolescent elite athlete populations, as, to our knowledge, such normative data are currently lacking in the literature.

Furthermore, there were inconsistent findings in female and male models regarding sports pressure and transdiagnostic factors which do not correspond with our expectations. However, these results do not invalidate the potential relevance of emotion regulation and self-esteem in both genders, but rather point to the fact that there are different pathways influencing DE. One is through a sport-specific factor (sports pressures) influencing mood and directly and indirectly affecting DE in males. Another pathway is taken by the transdiagnostic factors (such as emotion regulation and self-esteem), which are involved in a different set of pathways associated with DE in female athletes. It is also important to consider that some of the non-significant associations observed –particularly in the male subsample – may be influenced by methodological factors. The smaller sample size of male athletes could have reduced statistical power, especially in the context of a relatively complex structural model involving multiple latent variables and interrelated paths. When model complexity is high, smaller samples can lead to estimation challenges [72]. Notably, few studies have examined these relationships in female and male adolescent athletes in various sports. Further research is therefore needed to clarify these associations.

These findings have several practical implications for youth sport settings. Given that sociocultural pressures were strongly associated with body dissatisfaction and DE across genders, preventive interventions may be strengthened by explicitly addressing appearance-related expectations within teams and sports organizations. The gender-specific patterns identified in this study further suggest that interventions may require differentiated emphases: supporting emotion regulation and self-esteem may be particularly relevant for female athletes, whereas assisting male athletes managing sport-related performance pressures and negative mood may prove beneficial. Further, sport federations, coaches, and health professionals may use these insights to design age-appropriate and gender-informed prevention strategies that promote healthy body image and reduce vulnerability to DE in adolescent athletes.

### Limitations and future research

Several limitations should be considered when interpreting these findings. Firstly, all data were collected in a non-clinical sample, but the mean levels of DE in this sample of young athletes were like those found in a study of male adults (e.g., [73]), and even higher for female athletes. Therefore, we assume that in this sample, several participants are at risk of developing EDs. Further, the data were retrospective, self-reported, and cross-sectional, preventing causal inferences and limiting conclusions regarding the temporal sequence of these relationships. Secondly, the models have not yet been tested in an additional sample to allow exploration and confirmation of the model, limiting the generalizability of the findings. Thirdly, some of the questionnaires had to be translated for this study, and validation of the translated versions is missing. Fourthly, potential differences between types of sport (e.g., weight-sensitive sports and non-weight-sensitive sports) or level of sport (e.g., elite and non-elite athletes) were not examined due to sample size constraints and the use of different questionnaire versions depending on the athlete’s gender. This made it difficult to estimate a unified model across gender or to test the model separately by sport type or sport level. For these reasons, and because we aimed to evaluate a general model, we focused on testing the model separately for male and female athletes rather than by sport type or sport level. Fifthly, the “modeled behaviors” factor from Petrie and Greenleaf’s model was excluded from the present model due to the lack of validated quantitative measures [62]. Sixthly, some subscales, particularly in the male subsample, showed lower internal reliability. As lower reliability typically attenuates associations, significant effects in the model are likely to represent conservative estimates rather than inflated relationships. Seventhly, although Petrie and Greenleaf have proposed that self-esteem may function as a moderator within their model, we did not test moderation effects in the present study due to limited empirical evidence available to support this role. Future studies may benefit from examining whether self-esteem acts as a moderator in adolescent athletes, as this could further clarify how individual psychological factors contribute to vulnerability to DE. Future research should also address several additional aspects. First, DE might vary during the sports season and potentially increase during competition periods. A longitudinal study design could provide deeper insights into these variations and help to clarify the temporal ordering or potential bidirectional nature of the associations observed in the present model. Second, future research with larger samples could examine whether the extended model applies across sport types and competition levels. Finally, future studies may also explore whether developmental factors, such as age or training history, influence these associations.

## Conclusions

This study provides novel insights into how sociocultural and sport-specific factors are associated with DE in young male and female adolescent athletes. The findings emphasize the predominant role of sociocultural pressures in shaping body dissatisfaction and DE across genders, while also highlighting gender differences in influential factors: emotion regulation difficulties and low self-esteem were relevant in the female model, whereas sport weight pressures and negative mood were more salient in the male model. These results underscore the need for preventive interventions aimed at educating coaches and athletes on the impact of sociocultural and sport pressures, transdiagnostic factors increasing vulnerability, and negative mood, while promoting body acceptance to reduce the risk of DE.

## Supplementary Information

Supplementary Material 1.

Supplementary Material 2.

## Data Availability

Data supporting the findings of this study are available from the corresponding author, upon reasonable request.
