# Correction to: Psychosocial correlates of disordered eating among adolescent athletes: a cross-sectional study

**DOI:** 10.1186/s40337-026-01540-x

**Published:** 2026-02-02

**Authors:** Amandine Franzoni, Jean-Philippe Antonietti, Simone Munsch, Nadine Messerli-Bürgy

**Affiliations:** 1 FAmily and DevelOpment research center (FADO), Institute of Psychology, University of Lausanne, Lausanne, Switzerland; 2 Department of Psychology, Clinical Psychology and Psychotherapy, University of Fribourg, Fribourg, Switzerland; 3 Food Research and Innovation Center, FRIC, Cluster Food and Mental Health/Psychology, University of Fribourg, Fribourg, Switzerland

**Correction: J Eat Disord (2026) 14:8**

10.1186/s40337-025-01500-x.

Following the publication of the original article, the author reported that several parts required correction.

1. The approval of the study protocol was still masked in the original article. It should have read: “*The study protocol was approved by the ethics committee of the University of Lausanne*,* Switzerland (E_SSP_072023_00001)*,* and the study was conducted in accordance with the Declaration of Helsinki*.”
2. In several sections, x² is incorrectly written as x² 2, and in the section “Factors related to DE in young male athletes,” the chi‑square symbol is missing in the second paragraph. The correct sentence is (χ²(503) = ….

The original article has been corrected.

