# Supplementary material for: Psychosocial correlates of disordered eating among adolescent athletes: a cross-sectional study
Source: J Eat Disord. 2025 Dec 12;14:8. doi: 10.1186/s40337-025-01500-x (PMC12781391; doi:10.1186/s40337-025-01500-x)
Supplement: Supplementary file 1 — Supplementary Material 1. [file 40337_2025_1500_MOESM1_ESM.docx]

**Supplementary materials**

**R Script for creating subscales and parcels for the measurement model among female athletes**

#-------------------- Creating subscales -------------------------------#

#---Sport Pressure---#

#SP1 = Weight-pressure from coach/teammates/sport + weight limit

SportFData$SP1 <- rowMeans(SportFData[, c("WPS-F1", "WPS-F2","WPS-F3", "WPS-F4", "WPS-F8", "WPS-F12", "WPS-F13", "WPS-F14", "WPS-F15", "WPS-F16")], na.rm = TRUE)

#SP2 = self-consciousness of weight and appearance

SportFData$SP2 <- rowMeans(SportFData[, c("WPS-F5", "WPS-F6","WPS-F7", "WPS-F11")], na.rm = TRUE)

#---Sociocultural Pressure---#

#SC1 = Pressure - media

SportFData$SC1 <- rowMeans(SportFData[, c("SATAQF28", "SATAQF29", "SATAQF30", "SATAQF31")], na.rm = TRUE)

#SC2 = Pressure - peers and significant others

SportFData$SC2 <- rowMeans(SportFData[, c("SATAQF20", "SATAQF21", "SATAQF22", "SATAQF23", "SATAQF24", "SATAQF25", "SATAQF26", "SATAQF27")], na.rm = TRUE)

#SC3 = Pressure - family

SportFData$SC3 <- rowMeans(SportFData[, c("SATAQF16", "SATAQF17", "SATAQF18", "SATAQF19")], na.rm = TRUE)

#--- Internalization---#

#INT1 = Internalization - muscular

SportFData$INT1 <- rowMeans(SportFData[, c("SATAQF1", "SATAQF4", "SATAQF8", "SATAQF10R", "SATAQF15")], na.rm = TRUE)

#INT2 = Internalization - thin/low body fat

SportFData$INT2 <- rowMeans(SportFData[, c("SATAQF3", "SATAQF6", "SATAQF11", "SATAQF13")], na.rm = TRUE)

#INT3 = Internalization - general attractivness

SportFData$INT3 <- rowMeans(SportFData[, c("SATAQF2", "SATAQF5", "SATAQF7", "SATAQF9R", "SATAQF12", "SATAQF14R")], na.rm = TRUE)

#---Conformity---#

#C1 = Refuse to limit

SportFData$C1 <- rowMeans(SportFData[, c("CSES11", "CSES12", "CSES13", "CSES14", "CSES15", "CSES16", "CSES17R", "CSES18", "CSES19", "CSES20")], na.rm = TRUE)

#C2 = Self sacrifice without CSES10R, see Franzoni et al. submitted for publication

SportFData$C2 <- rowMeans(SportFData[, c("CSES7", "CSES8", "CSES9")], na.rm = TRUE)

#C3 = Strive for dinstinction

SportFData$C3 <- rowMeans(SportFData[, c("CSES1", "CSES2", "CSES3", "CSES4", "CSES5","CSES6")], na.rm = TRUE)

#---Athletic Identity---#

#AI1 = Social Identity

SportFData$AI1 <- rowMeans(SportFData[, c("AIMS1","AIMS2","AIMS3")], na.rm = TRUE)

#AI2 = Exclusivity

SportFData$AI2 <- rowMeans(SportFData[, c("AIMS4", "AIMS5")], na.rm = TRUE)

#AI3 = Negative affectivity

SportFData$AI3 <- rowMeans(SportFData[, c("AIMS6", "AIMS7")], na.rm = TRUE)

#--- Emotion regulation---#

#ER1 = Strategie

SportFData$ER1 <- rowMeans(SportFData[, c("DERS10", "DERS15", "DERS18")], na.rm = TRUE)

#ER2 = Non-acceptation

SportFData$ER2 <- rowMeans(SportFData[, c("DERS7", "DERS12", "DERS16")], na.rm = TRUE)

#ER3 = Impulsion

SportFData$ER3 <- rowMeans(SportFData[, c("DERS9", "DERS14", "DERS17")], na.rm = TRUE)

#ER4 = Objectif

SportFData$ER4 <- rowMeans(SportFData[, c("DERS8", "DERS11", "DERS13")], na.rm = TRUE)

#ER5 = Clarté

SportFData$ER5 <- rowMeans(SportFData[, c("DERS2", "DERS3", "DERS5")], na.rm = TRUE)

#ER6 = Conscience

SportFData$ER6 <- rowMeans(SportFData[, c("DERS1R", "DERS4R", "DERS6R")], na.rm = TRUE)

#--- Disordered eating behaviors - DEB ---#

#DEB sans shape and weight concern

#DEB1 = eating concern

SportFData$DEB1 <- rowMeans(SportFData[,c("EDEQ7", "EDEQ9", "EDEQ19", "EDEQ20", "EDEQ21")], na.rm=TRUE)

#DEB2 = restraint eating

SportFData$DEB2 <- rowMeans(SportFData[,c("EDEQ1", "EDEQ2", "EDEQ3", "EDEQ4", "EDEQ5")], na.rm=TRUE)

#DEB3 = compensatory behaviors

SportFData$DEB3 <- rowMeans(SportFData[,c("EDEQ13", "EDEQ14", "EDEQ15", "EDEQ16", "EDEQ17", "EDEQ18")],na.rm=TRUE)

#------------------------ Creating parcels ----------------------------------#

#---Negative mood---#

# Specify the indicators for parceling, I used the Item-to-construct Balance method (Little et al., 2002)

data_NM <- SportFData[,c("PHQ1", "PHQ2", "PHQ3", "PHQ4", "PHQ5","PHQ6","PHQ7","PHQ8","PHQ9")]

SportFData$NMp1 <- apply(data_MH[,c("PHQ2", "PHQ1","PHQ7")],1,mean,na.rm=TRUE)

SportFData$NMp2 <- apply(data_MH[,c("PHQ6", "PHQ9","PHQ3")],1,mean,na.rm=TRUE)

SportFData$NMp3 <- apply(data_MH[,c("PHQ5", "PHQ4","PHQ8")],1,mean,na.rm=TRUE)

#---Self Esteem---#

# Specify the indicators for parceling, I used the Item-to-construct Balance method (Little et al., 2002)

data_SE <- SportFData[,c("RSES1","RSES2","RSES3R","RSES4","RSES5R","RSES6","RSES7","RSES8R","RSES9R","RSES10R")]

SportFData$SEp1 <- apply(data_SE[,c("RSES7", "RSES1", "RSES4", "RSES8R")],1,mean,na.rm=TRUE)

SportFData$SEp2 <- apply(data_SE[,c("RSES6", "RSES2", "RSES5R")],1,mean,na.rm=TRUE)

SportFData$SEp3 <- apply(data_SE[,c("RSES10R", "RSES9R", "RSES3R")],1,mean,na.rm=TRUE)

#--- Body dissatisfaction ---#

# Creation of variables BD1 and BD2 using manathan distance.

#BD1 (self-ideal discrepancy ; item 1 - item 3):

DIST <- function(X1, X2){

n <- length(X1)

D <- NULL

for(i in 1:n){

u1 <- (X1[i] - 1) %/% 8

v1 <- (X1[i] - 1) %% 8

u2 <- (X2[i] - 1) %/% 8

v2 <- (X2[i] - 1) %% 8

d <- abs(u1 - u2) + abs(v1 - v2)

D <- c(D, d)

}

return(D)

}

cbind(X1 = SportFData$BIMTMFB1, X2 = SportFData$BIMTMFB3, D = DIST(SportFData$BIMTMFB1, SportFData$BIMTMFB3))

SportFData$BD1 <- DIST(SportFData$BIMTMFB1, SportFData$BIMTMFB3)

#BD2 (item 2 - item 3):

DIST <- function(X3, X4){

n <- length(X3)

D <- NULL

for(i in 1:n){

u1 <- (X3[i] - 1) %/% 8

v1 <- (X3[i] - 1) %% 8

u2 <- (X4[i] - 1) %/% 8

v2 <- (X4[i] - 1) %% 8

d <- abs(u1 - u2) + abs(v1 - v2)

D <- c(D, d)

}

return(D)

}

cbind(X3 = SportFData$BIMTMFB2, X4 = SportFData$BIMTMFB3, D = DIST(SportFData$BIMTMFB2, SportFData$BIMTMFB3))

SportFData$BD2 <- DIST(SportFData$BIMTMFB2, SportFData$BIMTMFB3)

#--- Drive for muscularity---#

#DM1 = Muscularity-oriented body image

SportFData$DM1 <- rowMeans(SportFData[, c("DMS1", "DMS7", "DMS9", "DMS11", "DMS13", "DMS14", "DMS15")], na.rm = TRUE)

#DM2 = Muscularity behavior

SportFData$DM2 <- rowMeans(SportFData[, c("DMS2", "DMS3", "DMS4", "DMS5", "DMS6", "DMS8", "DMS12")], na.rm = TRUE)

**R Script for creating the measurement model among female athletes**

Initial measurement model

M0 <- '

+

+ # latent variable definitions

+ SP =~ SP1 + SP2

+ SC =~ SC1 + SC2 + SC3

+ INT =~INT1 + INT2 + INT3

+ BD =~ BD1 + BD2

+ DMS =~ DM1 + DM2

+ DEB =~ DEB1 + DEB2

+ NM =~ NMp1 + NMp2 + NMp3

+ CONF =~ C1 + C2 + C3

+ AI =~ AI1 + AI2 + AI3

+ SE =~ SEp1 + SEp2 + SEp3

+ ER =~ ER1 + ER2 + ER3 + ER4 + ER5 + ER6

+ '

>

> f0 = sem(M0, data = SportFData, missing = "fiml", estimator = "MLR")

> summary(f0, standardized = TRUE, ci = TRUE, fit.measures = TRUE, rsquare=TRUE)

Final measurement model

M04 <- '

# latent variable definitions

SP =~ SP1 + SP2

SC =~ SC1 + SC2 + SC3

INT =~ INT2 + INT3

BD =~ 1*BD1 + 1*BD2

DMS =~ MBI1 + MBI2 + MBI3

DEB =~ DEB1 + DEB2

NM =~ NMp1 + NMp2 + NMp3

CONF =~ C1 + C2 + C3

AI =~ AI1 + AI2 + AI3

SE =~ SEp1 + SEp2 + SEp3

ER =~ ER1 + ER2 + ER3 + ER4 + ER5

'

f04 = sem(M04, data = SportFData, missing = "fiml", estimator = "MLR")

summary(f04, standardized = TRUE, ci = TRUE, fit.measures = TRUE, rsquare=TRUE)

**R Script for creating subscales and parcels for the measurement model among male athletes**

#-------------------- Creating subscales -------------------------------#

#---Sport Pressure---#

#SP1 = teammate pressure about weight

SportMData$SP1 <- rowMeans(SportMData[, c("WPS-M1", "WPS-M2", "WPS-M3", "WPS-M4", "WPS-M5", "WPS-M6")], na.rm = TRUE)

#SP2 = Pressure about weight and body due to uniform

SportMData$SP2 <- rowMeans(SportMData[, c("WPS-M7", "WPS-M8", "WPS-M9")], na.rm = TRUE)

#SP3 = Importance of body weight and appearance

SportMData$SP3 <- rowMeans(SportMData[, c("WPS-M10", "WPS-M11", "WPS-M12")], na.rm = TRUE)

#---Sociocultural Pressure---#

#SC1 = Pressure - media

SportMData$SC1 <- rowMeans(SportMData[, c("SATAQM23", "SATAQM24", "SATAQM25", "SATAQM26", "SATAQM27", "SATAQM28")], na.rm = TRUE)

#SC2 = Pressure - peers/family/significant

SportMData$SC2 <- rowMeans(SportMData[, c("SATAQM9", "SATAQM10", "SATAQM11", "SATAQM12", "SATAQM13", "SATAQM14", "SATAQM15", "SATAQM16", "SATAQM17", "SATAQM18", "SATAQM19", "SATAQM20", "SATAQM21", "SATAQM22")], na.rm = TRUE)

#--- Internalization---#

#INT1 = Internalization - muscular

SportMData$INT1 <- rowMeans(SportMData[, c("SATAQM1", "SATAQM3", "SATAQM5","SATAQM8")], na.rm = TRUE)

#INT2 = Internalization - thin/low body fat

SportMData$INT2 <- rowMeans(SportMData[, c("SATAQM2", "SATAQM4")], na.rm = TRUE)

#INT3 = Internalization - general attractivness

SportMData$INT3 <- rowMeans(SportMData[, c("SATAQM6R", "SATAQM7R")], na.rm = TRUE)

#--- Drive for muscularity---#

#DM1 = Muscularity-oriented body image

SportMData$DM1 <- rowMeans(SportMData[, c("DMS1", "DMS7", "DMS9", "DMS11", "DMS13", "DMS14", "DMS15")], na.rm = TRUE)

#DM2 = Muscularity behavior

SportMData$DM2 <- rowMeans(SportMData[, c("DMS2", "DMS3", "DMS4", "DMS5", "DMS6", "DMS8", "DMS12")], na.rm = TRUE)

#MBI1 = Muscularity-oriented body image (MBI) parcel 1

SportMData$MBI1 <- rowMeans(SportMData[, c("DMS9", "DMS11", "DMS13", "DMS14")], na.rm = TRUE)

#MBI2 = Muscularity-oriented body image (MBI) parcel 2

SportMData$MBI2 <- rowMeans(SportMData[, c("DMS1", "DMS7", "DMS15")], na.rm = TRUE)

#---Conformity---#

#C1 = Refuse to limit

SportMData$C1 <- rowMeans(SportMData[, c("CSES11", "CSES12", "CSES13", "CSES14", "CSES15", "CSES16", "CSES17R", "CSES18", "CSES19", "CSES20")], na.rm = TRUE)

#C2 = Self sacrifice

SportMData$C2 <- rowMeans(SportMData[, c("CSES7", "CSES8", "CSES9", "CSES10R")], na.rm = TRUE)

#C3 = Strive for dinstinction

SportMData$C3 <- rowMeans(SportMData[, c("CSES1", "CSES2", "CSES3", "CSES4", "CSES5","CSES6")], na.rm = TRUE)

#---Athletic Identity---#

#AI1 = Social Identity

SportMData$AI1 <- rowMeans(SportMData[, c("AIMS1","AIMS2","AIMS3")], na.rm = TRUE)

#AI2 = Exclusivity

SportMData$AI2 <- rowMeans(SportMData[, c("AIMS4", "AIMS5")], na.rm = TRUE)

#AI3 = Negative affectivity

SportMData$AI3 <- rowMeans(SportMData[, c("AIMS6", "AIMS7")], na.rm = TRUE)

#--- Emotion regulation---#

#ER1 = Strategie

SportMData$ER1 <- rowMeans(SportMData[, c("DERS10", "DERS15", "DERS18")], na.rm = TRUE)

#ER2 = Non-acceptation

SportMData$ER2 <- rowMeans(SportMData[, c("DERS7", "DERS12", "DERS16")], na.rm = TRUE)

#ER3 = Impulsion

SportMData$ER3 <- rowMeans(SportMData[, c("DERS9", "DERS14", "DERS17")], na.rm = TRUE)

#ER4 = Objectif

SportMData$ER4 <- rowMeans(SportMData[, c("DERS8", "DERS11", "DERS13")], na.rm = TRUE)

#ER5 = Clarté

SportMData$ER5 <- rowMeans(SportMData[, c("DERS2", "DERS3", "DERS5")], na.rm = TRUE)

#ER6 = Conscience

SportMData$ER6 <- rowMeans(SportMData[, c("DERS1R", "DERS4R", "DERS6R")], na.rm = TRUE)

#--- Disordered eating behaviors - DEB ---#

#DEB sans shape and weight concern

#DEB1 = eating concern

SportMData$DEB1 <- rowMeans(SportMData[,c("EDEQ7", "EDEQ9", "EDEQ19", "EDEQ20", "EDEQ21")], na.rm=TRUE)

#DEB2 = restraint eating

SportMData$DEB2 <- rowMeans(SportMData[,c("EDEQ1", "EDEQ2", "EDEQ3", "EDEQ4", "EDEQ5")], na.rm=TRUE)

#DEB3 = compensatory behaviors

SportMData$DEB3 <- rowMeans(SportMData[,c("EDEQ13", "EDEQ14", "EDEQ15", "EDEQ16", "EDEQ17", "EDEQ18")],na.rm=TRUE)

#------------------------ Creating parcels----------------------------------#

#---negative mood---#

# Specify the indicators for parceling, I used the Item-to-construct Balance method (Little et al., 2002)

SportMData$NMp1 <- rowMeans(SportMData[,c("PHQ2", "PHQ3","PHQ7")], na.rm=TRUE)

SportMData$NMp2 <- rowMeans(SportMData[,c("PHQ6", "PHQ8","PHQ4")], na.rm=TRUE)

SportMData$NMp3 <- rowMeans(SportMData[,c("PHQ5", "PHQ9","PHQ1")], na.rm=TRUE)

#---Self Esteem---#

# Specify the indicators for parceling, I used the Item-to-construct Balance method (Little et al., 2002)

SportMData$SEp1 <- rowMeans(SportMData[,c("RSES6", "RSES1", "RSES3R", "RSES5R")], na.rm=TRUE)

SportMData$SEp2 <- rowMeans(SportMData[,c("RSES9R", "RSES8R", "RSES7")], na.rm=TRUE)

SportMData$SEp3 <- rowMeans(SportMData[,c("RSES10R", "RSES4", "RSES2")], na.rm=TRUE)

#--- Body dissatisfaction ---#

#Creation of BD1 and BD2 variables using manathan distance.

#BD1 (self-ideal discrepancy ; item 1 - item 3):

DIST <- function(X1, X2){

n <- length(X1)

D <- NULL

for(i in 1:n){

u1 <- (X1[i] - 1) %/% 8

v1 <- (X1[i] - 1) %% 8

u2 <- (X2[i] - 1) %/% 8

v2 <- (X2[i] - 1) %% 8

d <- abs(u1 - u2) + abs(v1 - v2)

D <- c(D, d)

}

return(D)

}

cbind(X1 = SportMData$BIMTMMB1, X2 = SportMData$BIMTMMB3, D = DIST(SportMData$BIMTMMB1, SportMData$BIMTMMB3))

SportMData$BD1 <- DIST(SportMData$BIMTMMB1, SportMData$BIMTMMB3)

#BD2 (item 2 - item 3):

DIST <- function(X3, X4){

n <- length(X3)

D <- NULL

for(i in 1:n){

u1 <- (X3[i] - 1) %/% 8

v1 <- (X3[i] - 1) %% 8

u2 <- (X4[i] - 1) %/% 8

v2 <- (X4[i] - 1) %% 8

d <- abs(u1 - u2) + abs(v1 - v2)

D <- c(D, d)

}

return(D)

}

cbind(X3 = SportMData$BIMTMMB2, X4 = SportMData$BIMTMMB3, D = DIST(SportMData$BIMTMMB2, SportMData$BIMTMMB3))

SportMData$BD2 <- DIST(SportMData$BIMTMMB2, SportMData$BIMTMMB3)

**R Script for creating the measurement model among male athletes**

Initial measurement model

M0 <- '

# latent variable definitions

SP =~ SP1 + SP2 + SP3

SC =~ SC1 + SC2 + SC3 + SC4

INT =~ INT1 + INT2 + INT3

BD =~ BD1 + BD2

DMS =~ DM1 + DM2

DEB =~ DEB1 + DEB2 + DEB3

NM =~ NMp1 + NMp2 + NMp3

CONF =~ C1 + C2 + C3

AI =~ AI1 + AI2 + AI3

SE =~ SEp1 + SEp2 + SEp3

ER =~ ER1 + ER2 + ER3 + ER4 + ER5 + ER6

'

f0 = sem(M0, data = SportMData, missing = "fiml", estimator = "MLR")

summary(f0, standardized = TRUE, ci = TRUE, fit.measures = TRUE, rsquare=TRUE)

Final measurement model

M03b <- '

# latent variable definitions

SP =~ SP1 + SP2 + SP3

SC =~ SC1 + SC2 + SC3 + SC4

INT =~ INT1 + INT3

BD =~ BD1 + BD2

DMS =~ MBI1 + MBI2 + MBI3

DEB =~ DEB1 + DEB2 + DEB3

NM =~ NMp1 + NMp2 + NMp3

CONF =~ C1 + C2 + C3

AI =~ AI1 + AI2 + AI3

SE =~ SEp1 + SEp2 + SEp3

ER =~ ER1 + ER2 + ER3 + ER4 + ER5

ER3 ~~ ER4

'

f03b = sem(M03b, data = SportMData, missing = "fiml", estimator = "MLR")

summary(f03b, standardized = TRUE, ci = TRUE, fit.measures = TRUE, rsquare=TRUE)
