# Supplementary material for: Psychosocial correlates of disordered eating among adolescent athletes: a cross-sectional study
Source: J Eat Disord. 2025 Dec 12;14:8. doi: 10.1186/s40337-025-01500-x (PMC12781391; doi:10.1186/s40337-025-01500-x)
Supplement: Supplementary file 2 — Supplementary Material 2. [file 40337_2025_1500_MOESM2_ESM.docx]

**Table S1**

*Questionnaires and versions used for each factor of influence, and information about their reliability and validity*

| Influencing factor | Questionnaire | Version | Information on reliability | Information on validity |
| --- | --- | --- | --- | --- |
| Disordered eating behavior and Dietary restraint | Eating Disorder Examination Questionnaire (EDE-Q; original English version by Fairburn & Beglin, 1994) | French: version by Carrard et al. (2015) | α of the *eating concerns* and *restraint* subscales are .93 and .73, respectively (Turgeon et al., 2015). | Carrard et al. obtained good construct validity for a three-factor structure. |
|  |  | German: version by Hilbert et al. (2007) | α of the *eating concerns* and *restraint* subscales are .81 and .84, respectively (Hilbert et al., 2012). | Hilbert et al. (2012) obtained satisfactory psychometric properties for construct validity for a three-factor structure. |
|  |  | Italian: version by Calugi et al. (2016) | α of the *eating concerns* and *restraint* subscales are .79 and .80, respectively (Calugi et al., 2016). | Calugi et al. obtained good criterion validity and construct validity for a three-factor structure. |
| Sport weight pressures | Weight Pressure in Sport in female athlete (WPS-F; original English version by Reel et al., 2010) and Weight Pressure in Sport in male athlete (WPS-M; original English version by Galli et al., 2014) | French WPS-F: version by Porlier & Meilleur (2023)  French WPS-M: translation by Franzoni et al. | α of the *Weight pressures from coaches/team/sport/weight limits* and *self-consciousness of weight/appearance* subscales are .78 and .89, respectively (Porlier & Meilleur, 2023).  α of the *Weight pressures from coaches/team/sport/weight limits, self-consciousness of weight/appearance and experienced pressure regarding weight and body due to the sports uniform* subscales are .80, .73, and .52, respectively, while ω are .80, .74, and .58, respectively. | No available information.  The translated version has not yet been validated. |
|  |  | German and Italian WPS-F: translation by Franzoni et al.  German and Italian WPS-M: translation by Franzoni et al. | α of the *Weight pressures from coaches/team/sport/weight limits* and *self-consciousness of weight/appearance* subscales are .89 and .81, respectively, while ω are .89 and .83, respectively.  α of the *Weight pressures from coaches/team/sport/weight limits, self-consciousness of weight/appearance and experienced pressure regarding weight and body due to the sports uniform* subscales are .80, .73, and .52, respectively, while ω are .80, .74, and .58, respectively. | The translated versions has not yet been validated. |
| General sociocultural pressures and internalization of body ideal | Sociocultural Attitudes Towards Appearance Questionnaire-4 revised for female (SATAQ-4R-Female) and SATAQ-4R-Male (original English version by Schaefer et al., 2017) | French, German, and Italian SATAQ-4R-Female: translation by Franzoni et al.  French and German SATAQ-4R-Male: translation by Franzoni et al. | Female version: α of the seven subscales ranged from .82 to .94, and ω from .82 to .94  Male version: α of the seven subscales ranged from .81 to .95, and ω from .81 to .95 | The translated versions has not yet been validated. |
|  |  | Italian SATAQ-4R-Female and SATAQ-4R-Male: version by Stefanile et al. (2019) | Female version: α of the seven subscales ranged from .82 to .95  Male version: α of the seven subscales ranged from .80 to .97  (Stefanile et al., 2019) | Stefanile et al. provided evidence for the construct validity and convergent validity. |
| Body dissatisfaction | Body Image Matrix of Thinness and Muscularity – Female Bodies (BIMTM-FB; Steinfeld et al., 2020) and Body Image Matrix of Thinness and Muscularity – Male Bodies (BIMTM-MB; Arkenau et al., 2020) | | Test-retest reliability was high for both versions (Arkenau et al., 2020; Steinfeld et al., 2020). | Steinfeld et al. obtained good convergent validity for the female version, Arkenau et al. obtained good convergent and criterion validity for the male version. |
| Drive for muscularity | Drive for Muscularity Scale (DMS; original English version by McCreary & Sasse, 2000) | French: short version by Chaba et al. (2018) and partial translation by Franzoni et al. | α of the overall scale is .87 (Chaba et al., 2018). | Chaba et al. obtained good construct validity and sufficient concurrent validity. |
|  |  | German: version by Waldorf et al. (2014) | α of the overall scale is .90 (Waldorf et al., 2014). | Waldorf et al. obtained high convergent validity and a good construct validity. |
|  |  | Italian: version by Nerini et al. (2016) | α of the overall scale is .87 (Nerini et al., 2016). | Nerini et al. obtained good concurrent and construct validity. |
| Conformity | Conformity to the Sport Ethic Scale (CSES; original version in French by Parent et al., 2020). | French: version by Parent et al. (2020) | α of the *striving for distinction, self-sacrifice,* and *refusing to accept limits* subscales are .85, .72, and .76, respectively (Parent et al. 2020). | Parent et al. obtained good construct validity. |
|  |  | German and Italian: translation by Franzoni et al. | α of the three subscales ranged from .78 to .88, and ω from .79 to .88 (Franzoni et al., 2024). | Franzoni et al. obtained good construct validity for both translated version (manuscript submitted for publication). |
| Athletic identity | Athletic Identity Measurement Scale (AIMS; original English version by Brewer & Cornelius, 2001) | French: version by Caudroit et al. (2010) | α of the overall scale is .86 (Caudroit et al., 2010). | No available information. |
|  |  | German: version by Schmid & Seiler (2003) | α of the overall scale is .74 (Schmid & Seiler, 2003). | Schmid and Seiler obtained satisfactory psychometric properties for construct and convergent validity. |
|  |  | Italian: version by Costa et al. (2020) | α of the overall scale is .82 (Costa et al., 2020). | Costa et al. obtained good construct validity. |
| Self-esteem | Rosenberg Self-Esteem Scale (RSES; original English version by Rosenberg, 1965) | French: version by Vallières & Vallerand (1990) | α of the overall scale is .70 (Vallières & Vallerand, 1990). | Vallières & Vallerand obtained good construct and convergent validity. |
|  |  | German: version by Collani & Herzberg (2003) | α of the overall scale is .84 (Collani & Herzberg). | Collani & Herzberg obtained good construct validity. |
|  |  | Italian: version by Prezza et al. (1997) | α of the overall scale is .84 (Prezza et al., 1997). | Prezza et al. obtained construct validity. |
| Difficulties in emotional regulation | Difficulties in Emotion Regulation Scale – Short Form (DERS-SF; original English version by Gratz & Roemer, 2004) | French: version by Dan-Glauser & Scherer (2013) | α of the overall scale is .92 (Dan-Glauser & Scherer, 2013). | Dan-Glauser & Scherer obtained good construct validity. |
|  |  | German: version by Ehring et al. (2008) | α of the overall scale is .89 in the current study. | No available information. |
|  |  | Italian: version by Giromini et al. (2012) | α of the overall scale is .92 (Giromini et al., 2012). | Giromini et al. obtained good construct and concurrent validity. |
| Negative Mood | Patient Health Questionnaire-9 (PHQ-9; Spitzer et al., 1999) | French, German, and Italian: version by Spitzer et al. (1999) | α of the overall scale is .80 (Spitzer et al., 1999) | Spitzer et al. obtained good construct, criterion, and convergent validity. |

*Note*. The validity and reliability information for the translated versions by Franzoni et al. is based on the present data. α = Cronbach’s alpha. ω = Omega’s coefficient. All translated versions were translated according to the standards of Corbière and Fraccaroli (2020).

**References**

Arkenau, R., Vocks, S., Taube, C. O., Waldorf, M., & Hartmann, A. S. (2020). The Body Image Matrix of Thinness and Muscularity—Male Bodies : Development and validation of a new figure rating scale for body image in men. *Journal of Clinical Psychology*, *76*(7), 1283‑1292. <https://doi.org/10.1002/jclp.22933>

Brewer, B. W., & Cornelius, A. E. (2001). Norms and factorial invariance of the Athletic Identity Measurement Scale (AIMS). *The Academic Athletic Journal*, *15*, 103-113.

Calugi, S., Milanese, C., Sartirana, M., Ghoch, M. E., Sartori, F., Geccherle, E., Coppini, A., Franchini, C., & Grave, R. D. (2016). The Eating Disorder Examination Questionnaire : reliability and validity of the Italian version. *Eating And Weight Disorders - Studies on Anorexia Bulimia and Obesity*, *22*(3), 509‑514. <https://doi.org/10.1007/s40519-016-0276-6>

Carrard, I., Rebetez, M. M. L., Mobbs, O., & Van Der Linden, M. (2015). Factor structure of a French version of the eating disorder examination-questionnaire among women with and without binge eating disorder symptoms. *Eating And Weight Disorders - Studies on Anorexia Bulimia and Obesity*, *20*(1), 137‑144. <https://doi.org/10.1007/s40519-014-0148-x>

Caudroit, J., Stephan, Y., Brewer, B. W., & Scanff, C. L. (2010). Contextual and Individual Predictors of Psychological Disengagement From Sport During a Competitive Event. *Journal of Applied Social Psychology*, *40*(8), 1999‑2018. <https://doi.org/10.1111/j.1559-1816.2010.00648.x>

Chaba, L., D’Arripe-Longueville, F., Lentillon‐Kaestner, V., & Scoffier-Mériaux, S. (2018). Adaptation and validation of a short French version of the Drive for Muscularity Scale in male athletes (DMS-FR). *PLOS ONE*, *13*(5), 1-15. <https://doi.org/10.1371/journal.pone.0196608>

Collani, G. von, & Herzberg, P. Y. (2003). Zur internen Struktur des globalen Selbstwertgefühls nach Rosenberg. *Zeitschrift für Differentielle und Diagnostische Psychologie, 24*(1), 9–22. [https://doi.org/10.1024//0170-1789.24.1.9](https://doi.org/10.1024/0170-1789.24.1.9)

Corbière, M., & Fraccaroli, F. (2020). La conception, la validation, la traduction et l’adaptation transculuturelle d’outils de mesure : des exemples en santé mentale et travail. In M. Corbière & N. Larivière (Eds.), *Méthodes qualitatives, quantitatives et mixtes : dans la recherche en sciences humaines, sociales et de la santé* (2nd ed., pp. 703-752)*.* Presses de l’Université du Québec.

Costa, S., Santi, G., Di Fronso, S., Montesano, C., Di Gruttola, F., Ciofi, E. G., Morgilli, L., & Bertollo, M. (2020). Athletes and adversities : athletic identity and emotional regulation in time of COVID-19. *Sport Sciences for Health*, *16*(4), 609‑618. <https://doi.org/10.1007/s11332-020-00677-9>

Dan-Glauser, E. S., & Scherer, K. R. (2013). The Difficulties in Emotion Regulation Scale (DERS). *Swiss Journal of Psychology*, *72*(1), 5‑11. <https://doi.org/10.1024/1421-0185/a000093>

Ehring, T., Fischer, S., Schnülle, J., Bösterling, A., & Tuschen-Caffier, B. (2008). Characteristics of emotion regulation in recovered depressed versus never depressed individuals. *Personality and Individual Differences*, *44*(7), 1574‑1584. <https://doi.org/10.1016/j.paid.2008.01.013>

Fairburn, C. G., & Beglin, S. J. (1994). Assessment of eating disorders: Interview or self-report questionnaire? International Journal of Eating Disorders, 16(4), 363–370.

Franzoni, A., Meyer, A. H., Parent, S., & Messerli-Bürgy, N. (2024). *Validation of the Swiss German, Italian, and French version of the Conformity to the Sport Ethic Scale (CSES).* Manuscript submitted for publication.

Galli, N., Petrie, T. A., Reel, J. J., Chatterton, J. M., & Baghurst, T. M. (2014). Assessing the validity of the Weight Pressures in Sport Scale for Male Athletes. *Psychology of Men & Masculinity*, *15*(2), 170‑180. <https://doi.org/10.1037/a0031762>

Giromini, L., Velotti, P., De Campora, G., Bonalume, L., & Zavattini, G. C. (2012). Cultural Adaptation of the Difficulties in Emotion Regulation Scale : Reliability and Validity of an Italian Version. *Journal of Clinical Psychology*, *68*(9), 989‑1007. <https://doi.org/10.1002/jclp.21876>

Gratz, K. L., & Roemer, L. (2004). Multidimensional assessment of emotion regulation and dysregulation: Development, factor structure, and initial validation of the Difficulties in Emotion Regulation Scale. Journal of Psychopathology and Behavioral Assessment, 26(1), 41–54.

Hilbert, A., Tuschen-Caffier, B., Karwautz, A., Niederhofer, H., & Munsch, S. (2007). Eating Disorder Examination-Questionnaire. *Diagnostica*, *53*(3), 144‑154. <https://doi.org/10.1026/0012-1924.53.3.144>

Hilbert, A., De Zwaan, M., & Braehler, E. (2012). How Frequent Are Eating Disturbances in the Population ? Norms of the Eating Disorder Examination-Questionnaire. *PLoS ONE*, *7*(1), e29125. <https://doi.org/10.1371/journal.pone.0029125>

McCreary, D. R., & Sasse, D. K. (2000). An Exploration of the Drive for Muscularity in Adolescent Boys and Girls. *Journal of American College Health*, *48*(6), 297‑304. <https://doi.org/10.1080/07448480009596271>

Nerini, A., Matera, C., Baroni, D., & Stefanile, C. (2016). Drive for muscularity and sexual orientation : Psychometric properties of the Italian version of the Drive for Muscularity Scale (DMS) in straight and gay men. *Psychology of Men & Masculinity*, *17*(2), 137‑146. <https://doi.org/10.1037/a0039675>

Parent, S., Fortier, K., Vaillancourt-Morel, M., Lessard, G., Goulet, C., Demers, G., Paradis, H., & Hartill, M. (2020). Development and initial factor validation of the French Conformity to the Sport Ethic Scale (CSES). *Canadian Journal of Behavioural Science/Revue Canadienne des Sciences du Comportement*, *52*(4), 331‑336. <https://doi.org/10.1037/cbs0000168>

Porlier, G., & Meilleur, D. (2023). Attitudes et comportements face à l’image corporelle et l’alimentation chez un groupe d’adolescentes athlètes exerçant un sport de type esthétique à un haut niveau : l’influence des pressions de l’environnement sportif et de l’identité athlétique. *Neuropsychiatrie de L Enfance et de L Adolescence*, *71*(6), 325‑332. <https://doi.org/10.1016/j.neurenf.2023.03.007>

Prezza, M., Trombaccia, F. R., & Armento, L. (1997). La scala dell’autostima di Rosenberg: Traduzione e validazione italiana. *Bollettino di Psicologia Applicata, 223*, 35–44.

Reel, J. J., SooHoo, S., Petrie, T. A., Greenleaf, C., & Carter, J. E. (2010). Slimming down for sport: Developing a weight pressures in sport measure for female athletes. *Journal of Clinical Sport Psychology, 4*(2), 99–111.

Rosenberg, M. (1965). *Society and the adolescent self-image.* Princeton University Press.

Schaefer, L. M., Harriger, J. A., Heinberg, L. J., Soderberg, T., & Thompson, J. K. (2017). Development and validation of the sociocultural attitudes towards appearance questionnaire‐4‐revised (SATAQ‐4R). *International Journal of Eating Disorders*, *50*(2), 104‑117. <https://doi.org/10.1002/eat.22590>

Schmid, J., & Seiler, R. (2003). *Identität im Hochleistungssport: Psychometrische Untersuchungen mit einer deutschsprachigen Adaptation der Athletic Identity Measurement Scale (AIMS-D)*. *Diagnostica, 49*(4), 176–183. [https://doi.org/10.1026//0012-1924.49.4.176](https://doi.org/10.1026/0012-1924.49.4.176)

Spitzer, R. L., Kroenke, K., & Williams, J. B. W. (1999). Validation and utility of a self-report version of PRIME-MD: The PHQ primary care study. *JAMA, 282*(18), 1737–1744. <https://doi.org/10.1001/jama.282.18.1737>

Stefanile, C., Nerini, A., Matera, C., Schaefer, L. M., & Thompson, J. K. (2019). Validation of an Italian version of the Sociocultural Attitudes Towards Appearance Questionnaire-4-Revised (SATAQ-4R) on non-clinical Italian women and men. *Body Image*, *31*, 48‑58. <https://doi.org/10.1016/j.bodyim.2019.08.005>

Steinfeld, B., Hartmann, A. S., Waldorf, M., & Vocks, S. (2020). Development and initial psychometric evaluation of the Body Image Matrix of Thinness and Muscularity – Female Bodies. *Journal of Eating Disorders*, *8*(1). <https://doi.org/10.1186/s40337-020-00345-w>

Turgeon, M., Meilleur, D., & Blondin, S. (2015). Évaluation des attitudes et des comportements alimentaires : comparaison entre un groupe d’adolescentes athlètes pratiquant un sport esthétique et un groupe témoin. *Neuropsychiatrie de L’Enfance et de L’Adolescence*, *63*(3), 175‑182. <https://doi.org/10.1016/j.neurenf.2015.01.001>

Vallières, É. F., & Vallerand, R. J. (1990). *Traduction et validation canadienne-française de l'échelle de l'estime de soi de Rosenberg*. *International Journal of Psychology, 25*(3), 305–316. <https://doi.org/10.1080/00207599008247865>

Waldorf, M., Cordes, M., Vocks, S., & McCreary, D. (2014). „Ich wünschte, ich wäre muskulöser” : Eine teststatistische Überprüfung der deutschsprachigen Fassung der Drive for Muscularity Scale (DMS). *Diagnostica*, *60*(3), 140‑152. <https://doi.org/10.1026/0012-1924/a000106>
